# Supplementary material for: Expanding Canonical Spider Silk Properties through a DNA Combinatorial Approach
Source: Materials (Basel). 2020 Aug 14;13(16):3596. doi: 10.3390/ma13163596 (PMC7475873; doi:10.3390/ma13163596)
Supplement: Supplementary file 1 [file materials-13-03596-s001.pdf]

materials

MDPI

Supplemental Material

Expanding Canonical Spider Silk Properties through a DNA Combinatorial Approach

Zaroug Jaleel [1,2,†], Shun Zhou [1,3,†], Zaira Martín-Moldes [1], Lauren M. Baugh [1,4], Jonathan Yeh [1,5], Nina Dinjaski [1,6], Laura T. Brown [1,7], Jessica E. Garb [8] and David L. Kaplan [1,*]

1 Department of Biomedical Engineering, Tufts University, 4 Colby St, Medford, MA 02155, USA; zarougj@bu.edu (Z.J.); shun.zhou@genscript.com (S.Z.); Zaira.Martin_Moldes@tufts.edu (Z.M.-M.); lbaugh@mit.edu (L.M.B.); yeh.jona@gmail.com (J.Y.); ndinjaski@partners.org (N.D.); Laura.Brown@milliporesigma.com (L.T.B.)
2 School of Medicine, Boston University, Boston, MA 02118, USA
3 National Engineering Laboratory for Modern Silk, College of Textile and Clothing Engineering, Soochow University, Suzhou 215123, China
4 Department of Biological Engineering, Massachusetts Institute of Technology, 21 Ames St #56-651, Cambridge, MA 02142, USA
5 Department of Process Development, Akouos Inc., 645 Summer St. Boston, MA 02210, USA
6 Division of Innovation, Partners HealthCare Innovation, 215 First Street, Cambridge, MA 02142, USA
7 Department of Research Solutions North America, MilliporeSigma, 400 Summit Dr, Burlington, MA 01803, USA
8 Department of Biological Science, University of Massachusetts Lowell, 198 Riverside Street, Olsen Hall 234, Lowell, MA 01854, USA; Jessica_Garb@uml.edu
* Correspondence: david.kaplan@tufts.edu
† These authors contributed equally to this work

Received: 6 July 2020; Accepted: 10 August 2020; Published: 14 August 2020

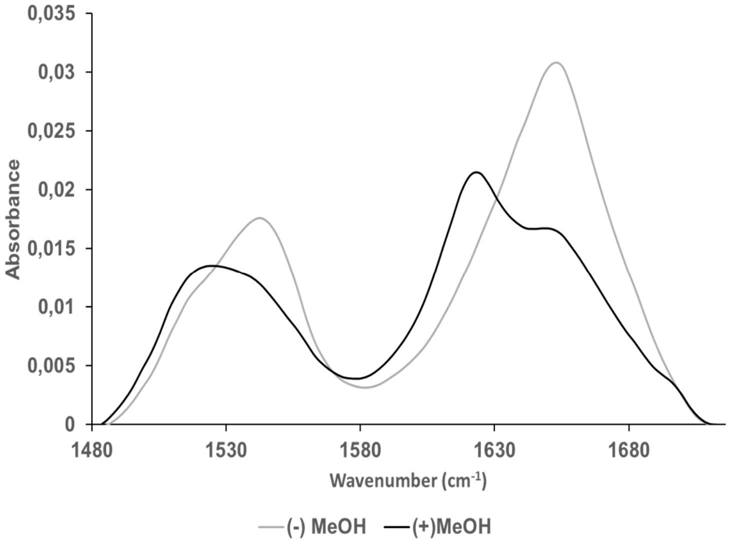

Figure S1. Representative FTIR spectra for recombinant A10$_2$ across amide I and amide II absorption spectra with methanol treated (black) and untreated (gray) films.

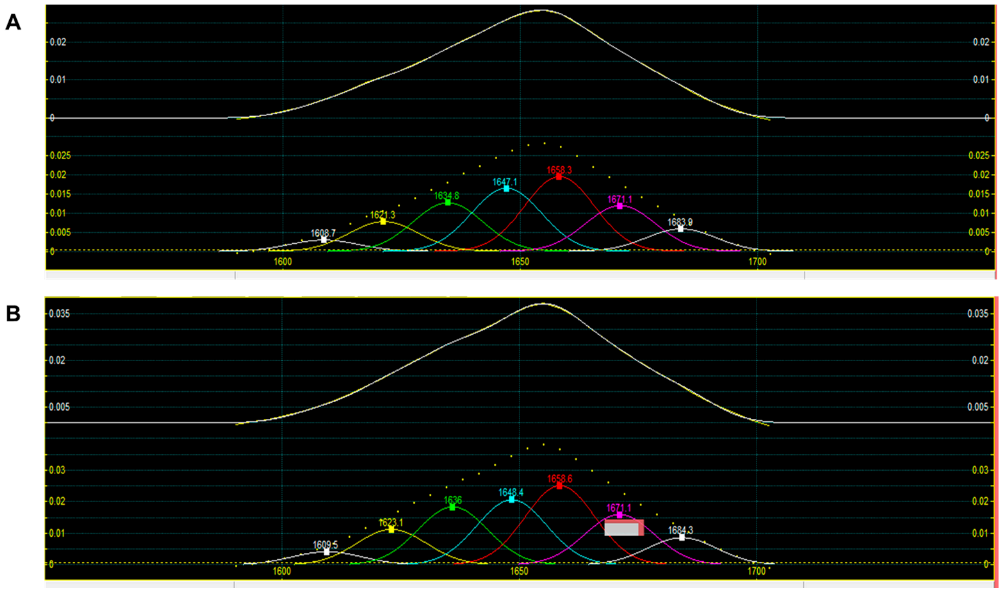

Figure S2. Representative deconvolution peaks for recombinant A10$_2$ across amide I and amide II absorption spectra of untreated (**A**) and methanol treated (**B**) films. Gaussian peaks were generated using PeakFit software included in the SigmaPlot package.

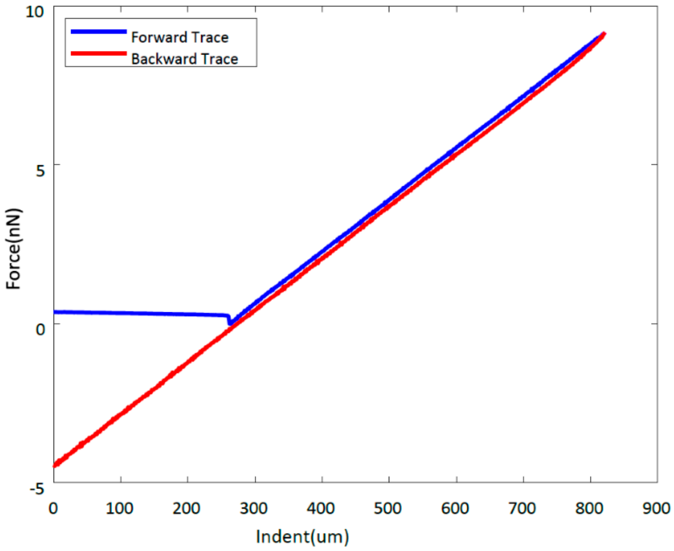

Figure S3. Representative load-displacement trace–retrace curve for recombinant A10$_2$ methanol treated.

Table S1. Measurement of surface roughness of the films generated before or after methanol treatment (MeOH) for A261, A102 and B10-22-17$_2$ protein constructs.

Protein Construct	Roughness (RMS; nm)	SD (nm)
A261	339	83
A261 MeOH	295	78
A10$_2$	166	61
A10$_2$ MeOH	165	51
B10-22-17$_2$	59	29
B10-22-17$_2$ MeOH	156	30

© 2020 by the authors. Licensee MDPI, Basel, Switzerland. This article is an open access article distributed under the terms and conditions of the Creative Commons Attribution (CC BY) license (http://creativecommons.org/licenses/by/4.0/).
